# CDK7 Inhibition Is Effective in all the Subtypes of Breast Cancer: Determinants of Response and Synergy with EGFR Inhibition

**DOI:** 10.3390/cells9030638

**Published:** 2020-03-06

**Authors:** Martina S. J. McDermott, Amanda C. Sharko, Jessica Munie, Susannah Kassler, Theresa Melendez, Chang-uk Lim, Eugenia V. Broude

**Affiliations:** Department of Drug Discovery and Biomedical Sciences, South Carolina College of Pharmacy, University of South Carolina, Columbia, SC 29208, USA; mmcdermott@mednet.ucla.edu (M.S.J.M.); SHARKO@cop.sc.edu (A.C.S.); jessmunie@comcast.net (J.M.); susannah146@gmail.com (S.K.); theresamelendez13@gmail.com (T.M.); limc@cop.sc.edu (C.-u.L.)

**Keywords:** CDK7, breast cancer, transcription, CITED2, EGFR

## Abstract

CDK7, a transcriptional cyclin-dependent kinase, is emerging as a novel cancer target. Triple-negative breast cancers (TNBC) but not estrogen receptor-positive (ER+) breast cancers have been reported to be uniquely sensitive to the CDK7 inhibitor THZ1 due to the inhibition of a cluster of TNBC-specific genes. However, bioinformatic analysis indicates that CDK7 RNA expression is associated with negative prognosis in all the major subtypes of breast cancer. To further elucidate the effects of CDK7 inhibition in breast cancer, we profiled a panel of cell lines representing different breast cancer subtypes. THZ1 inhibited cell growth in all subtypes (TNBC, HER2+, ER+, and HER2+/ER+) with no apparent subtype selectivity. THZ1 inhibited CDK7 activity and induced G1 arrest and apoptosis in all the tested cell lines, but THZ1 sensitivity did not correlate with CDK7 inhibition or CDK7 expression levels. THZ1 sensitivity across the cell line panel did not correlate with TNBC-specific gene expression but it was found to correlate with the differential inhibition of three genes: CDKN1B, MYC and transcriptional coregulator CITED2. Response to THZ1 also correlated with basal CITED2 protein expression, a potential marker of CDK7 inhibitor sensitivity. Furthermore, all of the THZ1-inhibited genes examined were inducible by EGF but THZ1 prevented this induction. THZ1 had synergistic or additive effects when combined with the EGFR inhibitor erlotinib, with no outward selectivity for a particular subtype of breast cancer. These results suggest a potential broad utility for CDK7 inhibitors in breast cancer therapy and the potential for combining CDK7 and EGFR inhibitors.

## 1. Introduction

In recent years, the advent of targeted therapies for estrogen receptor positive (ER+) and human epidermal growth factor receptor 2 positive (HER2+) breast cancers has significantly improved outcomes for breast cancer patients. However, innate treatment resistance and high rates of relapse following treatment are significant clinical issues in the management of breast cancer. In addition, there is a distinct lack of targeted therapies and treatment options for triple negative breast cancer (TNBC).

A novel treatment strategy currently under investigation involves targeting transcriptional regulation via inhibition of a subclass of cyclin dependent kinases that are involved primarily in transcription rather than cell cycle progression [1,2,3,4]. Phosphorylation of serines in the heptapeptide repeat of the carboxy-terminal domain of RNA polymerase II (RNA Pol II) is an essential step for efficient transcription. Cyclin dependent kinase 7 (CDK7) functions as part of the human transcription factor II (TFIIH) complex that phosphorylates RNA Pol II at serine 5 and serine 7 during initiation and promoter clearance, allowing the elongation complex to move downstream from the transcriptional start site [5,6]. In addition to regulating transcription, CDK7 can promote cell cycle progression by acting as a CDK-activating kinase (CAK) in a trimeric complex with Cyclin H and MAT1, phosphorylating cell cycle CDKs such as CDK1, CDK2, CDK4, and CDK6 [7,8]. Thus, inhibition of CDK7 offers a unique opportunity to target transcription and cell cycle progression simultaneously.

Interest in targeting CDK7 for the treatment of cancer has been boosted by the development of a potent covalent inhibitor, THZ1 [9]. In preclinical studies, THZ1 showed promising results in T-cell leukemia [9], neuroblastoma [10], small cell lung cancer [11], glioma [12] and ovarian cancer [13]. CDK7 may also serve as a novel druggable target for multiple subtypes of breast cancer. CDK7, Cyclin H, and MAT1 protein and mRNA expression were found to be upregulated in breast cancer tumors, compared to matched adjacent normal breast tissue, particularly in ER+ breast cancer [14]. Moreover, CDK7 expression positively correlated with ER expression and ER phosphorylation at S118, a site critical for ER-driven transcription and a known target of CDK7 activity [14,15], and THZ1 attenuates estradiol-induced phosphorylation of ER at S118 in vitro [16]. Another study has shown that HER2+ breast cancers may be more sensitive to CDK7 inhibition than ER+ breast cancers and that THZ1 may restore lost sensitivity to HER2-targeting therapies [17]. Of special interest, CDK7 has been identified as a promising therapeutic target in TNBC, which was reported to be especially sensitive to THZ1 among the principal molecular subtypes of breast cancer [2,17].

Utilizing CRISPR-Cas9-mediated knockout of CDK7 and THZ1, TNBC was reported to be uniquely dependent on CDK7, in contrast to ER-positive breast cancers. This dependence was ascribed to an “Achilles cluster” of TNBC-specific critical genes regulated by super-enhancers requiring CDK7. THZ1 induced apoptosis in TNBC cell lines in vitro and inhibited the growth of cell-line and patient-derived xenograft models of TNBC in vivo [2]. The relevance of CDK7 as a TNBC target was further confirmed by a recent study by Li et al., revealing that elevated CDK7 mRNA and protein levels correlated with poor patient prognosis in TNBC. This study also showed that combining BCL-2/BCL-XL inhibitors with THZ1 resulted in synergistic growth inhibition and apoptosis in TNBC cells [18].

In an effort to reconcile the various findings detailed above, we explored the therapeutic potential of CDK7 inhibition in a panel of breast cancer cell lines representing the major breast cancer subtypes. We found THZ1 to be differentially effective across ER+, HER2+, and TNBC cell lines; however, there was no apparent selectivity for a particular subtype. We also explored the effects of CDK7 inhibition on transcription to identify potential biomarkers of response to THZ1 across different subtypes of breast cancer. Here we confirm that THZ1 decreases expression of a number of transcriptionally important genes in TNBC; however, these genes were also downregulated by THZ1 in ER+ and HER2+ cell lines, with no detectable relationship between subtype and magnitude of downregulation. We did find a significant correlation between THZ1 sensitivity and THZ1-induced decreases in gene expression. Furthermore, basal protein expression of one of these genes, CITED2, correlated with THZ1 sensitivity, suggesting that CITED2 may serve as a possible biomarker of response to CDK7 inhibitors in the treatment of all breast cancer subtypes. We also found that erlotinib, an EGFR dual inhibition inhibitor, and THZ1 produce synergistic effects in both erlotinib-sensitive and insensitive cell lines, suggesting that CDK7 may be a useful target for enhancement of EGFR-targeting drugs.

## 2. Materials and Methods

### 2.1. Kaplan–Meier Plotter Online Survival Analysis

Kaplan Meier relapse-free survival curves for CDK7 (211297_s_at) were generated using Kaplan Meier Plotter from a total of 3951 breast cancers (November 2019 dataset) of Affymetrix microarray data [19].

### 2.2. Cell Lines and Reagents

MDA-MB-468, MDA-MB-231, MCF7, T47D, BT474 and ZR-75-1 cells were from the American Type Culture Collection (Manassas, VA); all other cell lines were a gift from Dr. Norma O’Donovan (Dublin City University, Dublin, Ireland). MCF7 cells were maintained in DMEM-high glucose media (ThermoFisher Scientific, Waltham, MA) with 10% fetal bovine serum (FBS) (Atlanta Biologics, Flowery Branch, GA), 1% penicillin-streptomycin and 2 mM L-glutamine (VWR, Radnor, PA), 1 mM sodium pyruvate (Sigma-Aldrich, St Louis, MO) and 5 mg insulin (Sigma-Aldrich). MDA-MB-468, MDA-MB-231, SKBR3 and JIMT-1 cells were maintained in DMEM-high glucose media (ThermoFisher Scientific) with 10% FBS, 1% penicillin-streptomycin and 2 mM L-glutamine. All other cell lines were maintained in RPMI-1640 (ThermoFisher Scientific) with 10% FBS, 1% penicillin-streptomycin and 2mM L-glutamine. All cell lines were routinely confirmed to be free of Mycoplasma (MycoAlert PLUS mycoplasma detection kit (Lonza, Alpharetta, GA)) and were authenticated by STR profiling by the University of Arizona Genetic Core (Tuscon, AZ) or by Source Bioscience (Santa Fe Springs, CA) in 2016. THZ1 was purchased from MedChem Express (Monmouth Junction, NJ) as a 10 mM stock solution in DMSO. Erlotinib was purchased from LC Laboratories (Woburn, MA).

### 2.3. Western Blotting

Cells were plated in 100 mm^2^ petri dishes at a density of 1 × 10^6^ cells/plate and allowed to grow for 3–4 days until the cells reached 80% confluency. Cells were treated with THZ1 or with vehicle control (DMSO) prior to lysing cells in RIPA buffer containing protease inhibitor cocktail and PMSF. Protein concentration was determined using BCA assay (Pierce, ThermoFisher Scientific). All lysates were prepared in biological duplicates. Protein (50 µg) was resolved on 4–20% Express-Plus PAGE gels in Tris-MOPS (SDS) running buffer (GenScript, Piscataway, NJ), transferred to PVDF membranes and incubated at 4 °C overnight with primary antibodies: CDK7 (sc-529, Santa Cruz Biotechnology, Dallas, TX), ER (sc-543, Santa Cruz), HER2 (#4290, Cell Signaling Technology, Danvers, MA), RNA-Pol II-S2P (C152000005, Diagenode, Denville, NJ), RNA-Pol II-S5P (C152000007, Diagenode), RNA-Pol II-S7P (04-1570, Millipore Sigma, Burlington, MA) and RNA-Pol II (sc-900X, Santa Cruz), CITED2 (MAB5005, R+D Systems, Minneapolis, MN), Tubulin (T-9026, Millepore Sigma), followed by anti-rabbit (#31460, ThermoFisher Scientific), anti-mouse (31430, ThermoFisher Scientific) or anti-rat (AP136P, Millipore Sigma) secondary antibodies. Bands were visualized with Western Lighting Plus ECL detection reagent (Perkin Elmer, Waltham, MA) using ChemiDoc Touch™ Imaging System (Bio-Rad, Hercules, CA). Images were analyzed and densitometry performed using ImageLab software Ver 5.2.1 (Bio-Rad).

### 2.4. RNA Extraction, Reverse Transcription and q-PCR

Cells were plated in 100 mm^2^ petri dishes at a density of 1 × 10^6^ cells/plate and allowed to grow for 3–4 days until the cells reached 80% confluency. Cells were treated with THZ1 or with vehicle control (DMSO). Total RNA was extracted using RNAeasy Mini Kit (Qiagen, Germantown, MD) and 1 µg of total RNA was used to generate cDNA using iScript cDNA synthesis kit (Bio-Rad). Gene expression was quantified using iTaq Universal SYBR green super mix (Bio-Rad) on a CFX384 Real-Time PCR Detection System(Bio-Rad). Primers used for real-time PCR are listed in Table S1.

### 2.5. Cell Proliferation Assays

Cells were seeded in 12-well plates at densities ranging from 10,000–30,000 cells/well and after 24 h cells were treated with 10, 40, and 100 nM THZ1 in triplicate. After 7 days, the cells were imaged using Zeiss (Dublin, CA) Axiovert 200 microscope using a 20× objective lens. Additionally, cells were plated in 96-well plates at a density of 2000 cells/well and after 24 h treated with 0–10 µM THZ1. After 2 and 7 days the growth of the cells was measured by MTT assay.

### 2.6. Cell Cycle Analysis

MDA-MB-231 and T47D cells were seeded into 6-well plates and allow to grow to 60% confluency. Cells were washed with PBS and the media was replaced with serum-free media overnight. After serum starvation, cells were treated with serum-containing media in the presence or absence of 100 and 250 nM THZ1 for 24 h and 48 h. Cells were collected, fixed in ethanol overnight and cell cycle distribution was measured using DAPI staining on the LSR2 flow cytometer ( Becton-Dickinson, Franklin Lakes, NJ).

### 2.7. EGF Stimulation

SKBR3 cells were seeded into duplicate 6-well plates and allow to grow until 80% confluent. The cells were then washed with PBS and the media was replaced with serum-free media overnight. After serum starvation the cells were treated with 100 ng/mL EGF with or without 250 nM THZ1 for 6 h then lysates were collected for both RNA and protein analysis.

### 2.8. Synergy Analysis

Cells were seeded into 96-well plates and after 24 h plates were treated with THZ1 (0–50 nM), and erlotinib (0–5 µM) alone or together in fixed-ratio combinations. After 7 days cell proliferation was measured by MTT assay.

### 2.9. Statistical Analysis

All results were presented as mean ± standard deviation of 4–8 parallel assessments. Similar results were obtained from a minimum of two independent experiments. Statistical significance was tested using two-sided Student T-tests and populations were considered significantly different at *P* < 0.05. Densitometry was performed on duplicate immunoblots using ImageLab software and normalized to tubulin loading controls and then correlated with the THZ1 IC_50_ values of each cell line. Statistical analyses were performed using SPSS 18.0 (SPSS Inc, Chicago, IL). Bi-variant scatter graphs and Spearman rank analyses were performed to evaluate associations between protein levels, mRNA and response to inhibition. IC_50_ values were calculated for MTT assays using CompuSyn software [20].

## 3. Results

### 3.1. High CDK7 Expression is Associated with Worse Relapse Free Survival in Breast Cancer Subtypes

**Figure 1:**
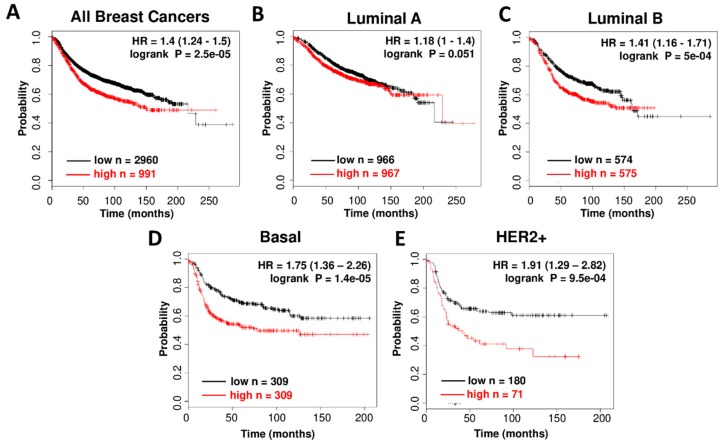
CDK7 RNA expression in breast cancer patient tumor samples. Association of CDK7 RNA expression with Relapse Free Survival (RFS) in microarray data from 3,951 breast cancer patient samples in all breast cancer patients (**A**), luminal-A patients (**B**), luminal-B patients (**C**), basal patients (**D**), and HER2+ patients (**E**), determined using KM-plotter online survival analysis tool [19].

Previous studies have reported that high CDK7 expression, together with Cyclin H and MAT1, was associated with better prognosis in ER-positive breast cancer patients [21] and worse prognosis in triple negative breast cancer patients [18]. We investigated correlations between CDK7 RNA expression and relapse-free survival (RFS) in breast cancer using a microarray database of 3,951 breast cancer patients. Kaplan-Meier (KM) plots show that high CDK7 expression is associated with worse Relapse Free Survival (RFS) in an unselected cohort of breast cancer patients representing multiple different subtypes of breast cancer (*p* =2.5 × 10^−05^, HR = 1.40) (Figure 1A). We then extended this analysis to examine correlations between CDK7 expression and RFS in the following breast cancer subtypes: luminal A, luminal B, basal and HER2 positive. High CDK7 RNA levels correlated with worse RFS for all breast cancer patients (Figure 1A), with the strongest associations found in basal (*p* = 1.4 × 10^−05^, HR = 1.75) and HER2+ (*p* = 9.5 × 10^−05^, HR = 1.91) subgroups (Figure 1D–E). This suggests that CDK7 may be an important novel target for breast cancer treatment for all breast cancer subtypes.

### 3.2. Breast Cancer Growth is Dependent on CDK7 Regardless of Subtype

**Figure 2:**
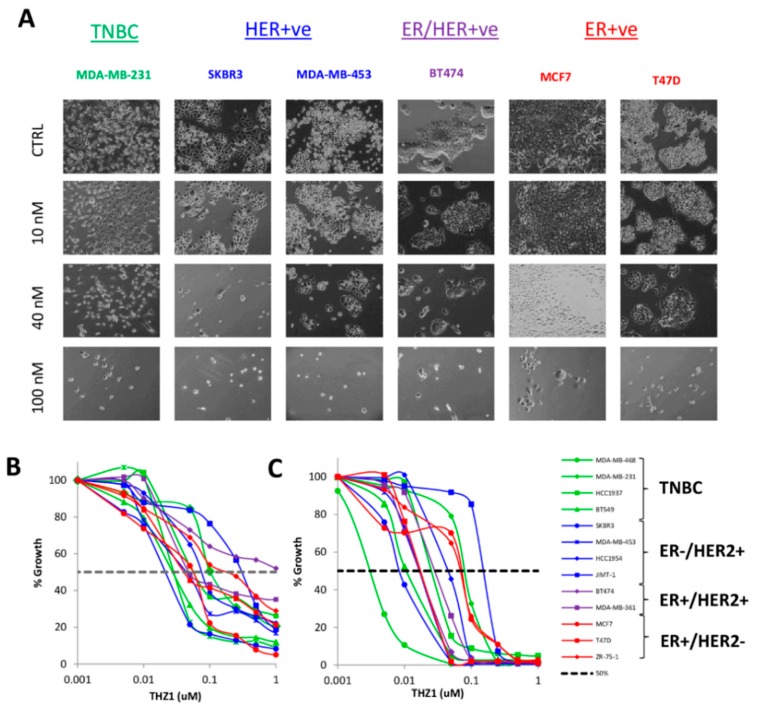
THZ1 inhibits the growth of breast cancer cell lines. (**A**) Bright-field images of cells treated with vehicle or 10, 40 and 100 nM of THZ1 for 7 days. Cell growth curves of TNBC (green), HER2-positive (blue), ER-positive/HER2-positive (purple), ER-positive/HER2-negative (red) breast cancer cell lines treated with increasing concentrations of THZ1 for (**B**) 2 days and (**C**) 7 days.

To explore the role of CDK7 in breast cancer growth, we first examined the effects of THZ1 on breast cancer cell lines encompassing TNBC, ER+/HER2-, ER+/HER2+ and ER-/HER2+ subtypes over seven days of treatment. While subtle differences in growth inhibition were observed at lower concentrations of THZ1, 100nM THZ1 inhibited the growth of all tested cell lines regardless of subtype (Figure 2A). To further investigate the effects of CDK7 inhibition on cell growth in different subtypes of breast cancer, we screened a panel of 13 breast cancer cell lines for response to THZ1 after 2 or 7 days of treatment. Two-day treatment with THZ1 (concentrations up to 1 µM) significantly inhibited cell growth, with most cell lines exhibiting IC_50_ values in the 80–300 nM range (Figure 2B, Table 1). Following 7 days of treatment, the only cell line exhibiting a lack of significant response to low nanomolar concentrations (<100nM) is JIMT-1 (Figure 2C, Table 1). There was a 25-fold difference in sensitivity at 7 days, as determined by IC_50_, between the most sensitive cell line, SKBR3 and the least sensitive cell line JIMT-1 (both of which are ER-/HER2+).

### 3.3. CDK7, ER and HER2 Expression are not Predictive Biomarkers of THZ1 Response

**Figure 3:**
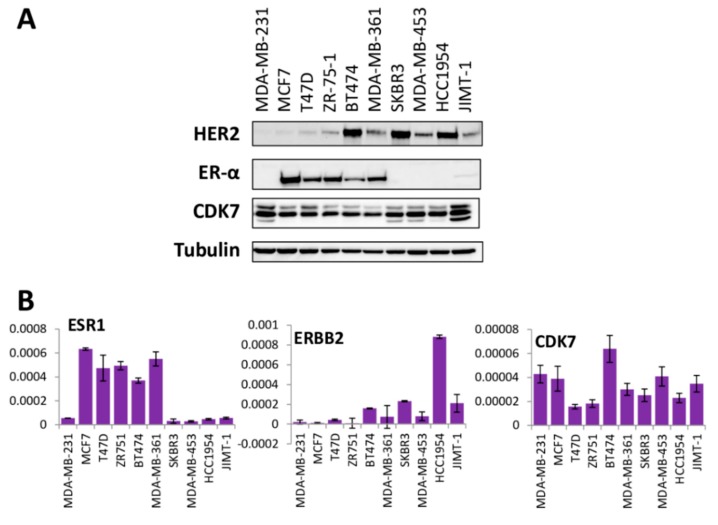
ER, HER2 and CDK7 expression in breast cancer cell lines. (**A**) Immunoblotting and (**B**) mRNA analysis of ERα (ESR1), HER2 (ERBB2) and CDK7 expression in a panel of cell lines representing multiple subtypes of breast cancer. mRNA represented as mRNA expression level normalized to GAPDH mRNA expression in sample.

**Figure 4:**
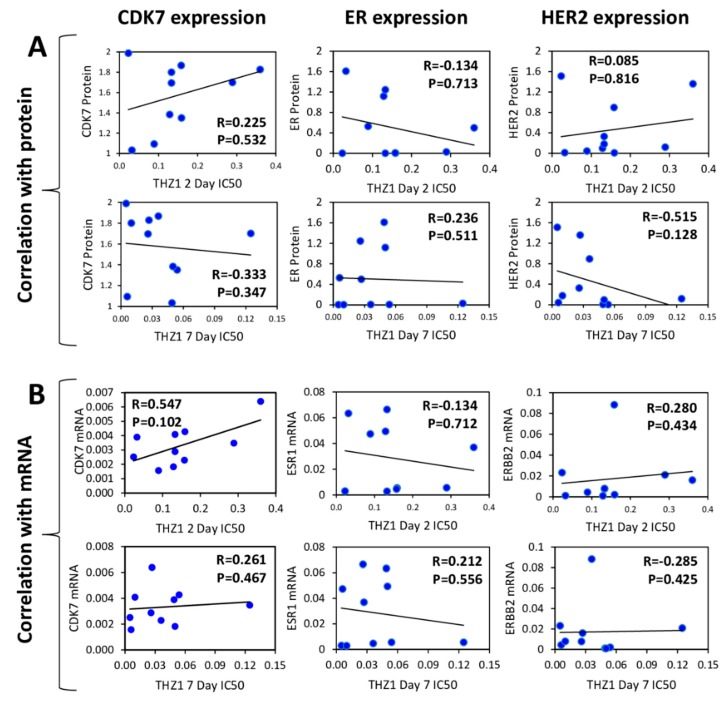
Correlations between CDK7, ER and HER2 expression in breast cancer cell line panel Bi-variant scattergraphs and Spearman rank correlations for CDK7, ER (ESR1) and HER2 (ERBB2) protein expression (**A**) and mRNA gene expression (**B**) levels compared to THZ1 2-day and 7-day IC_50_ values across the cell line panel. Protein levels expressed as mean densitometric measurements normalized to α-tubulin protein expression on the same blot. mRNA levels expressed as mean relative expression (determined by RT-qPCR) normalized to GAPDH mRNA expression in the same sample.

**Figure 5:**
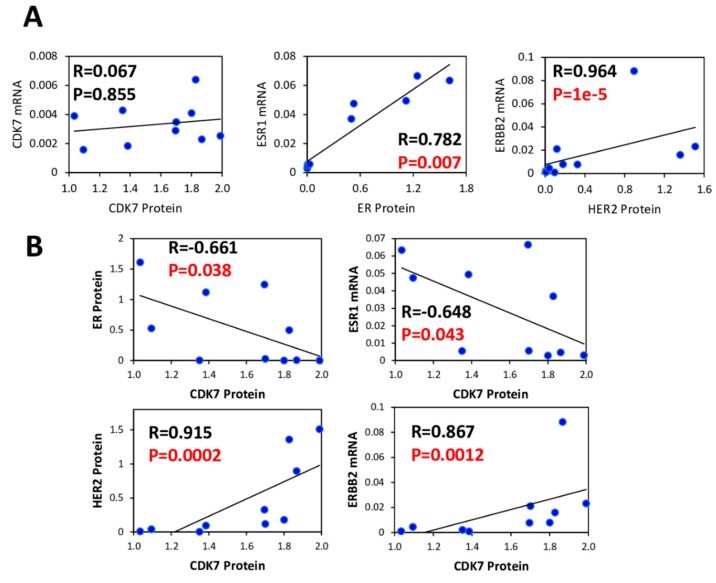
HER2 and ER are not markers of response to THZ1. (**A**) Bi-variant scattergraphs and Spearman rank correlations between CDK7, ER (ESR1) and HER2 (ERBB2) protein and mRNA expression levels (**B**) Bi-variant scattergraphs and Spearman rank correlations for CDK7 protein compared to protein and mRNA expression of ER (ESR1) or HER2 (ERBB2) across the cell line panel.

To determine if CDK7 expression levels were predictive of response to CDK7 inhibition, we measured protein and mRNA gene expression levels of CDK7, HER2 and ER in multiple breast cancer cell lines (Figure 3A and 3B). Neither CDK7 protein nor mRNA levels correlated with THZ1 sensitivity at either 2 days or 7 days of treatment (Figure 4A and B). Remarkably, there was no correlation between CDK7 protein and mRNA levels among the cell lines tested. In contrast, there were strong positive correlations between mRNA expression and protein expression levels for both HER2 and ERα (ESR1) (Figure 5A). We also found a weak inverse correlation between CDK7 protein expression and ESR1 mRNA expression and a strong positive correlation between CDK7 protein expression and both protein and mRNA expression levels of HER2 (Figure 5B). However, the differential expression of HER2 or ER was not predictive of response to CDK7 inhibition (Figure 4). These results suggest that the expression of CDK7, HER2 and ER are not predictive of response to CDK7 inhibition.

### 3.4. Phenotypic Responses to CDK7 Inhibition

**Figure 6:**
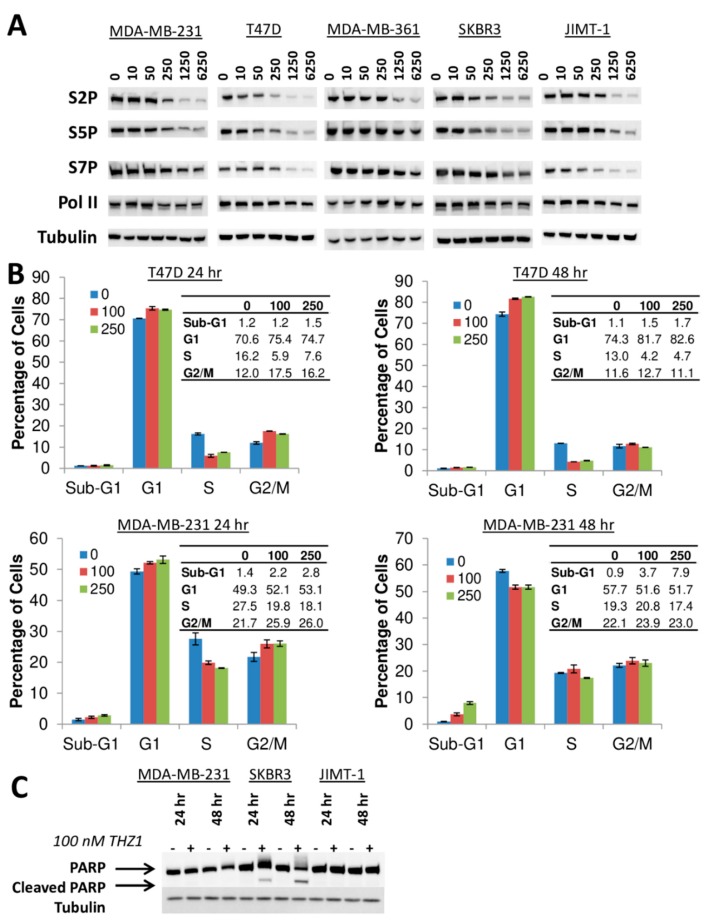
Effects of THZ1 (nM concentrations) on phosphorylation of RNA Pol II, cell cycle and apoptosis. (**A**) Immunoblotting for expression of phosphorylated RNA Pol II (S2P, S5P, S7P) and total Pol II protein (with α-tubulin as loading control) following treatment with increasing concentrations of THZ1 (0–6250 nM) for 4 h. (**B**) Cell cycle distribution profile for T47D and MDA-MB-231 cells treated with 100 or 250 nM THZ1 at 24 and 48 h. (**C**) Immunoblotting for PARP cleavage in MDA-MB-231, SKBR3 and JIMT-1 cells treated with 100 nM THZ1 for 24 or 48 h.

**Figure 7:**
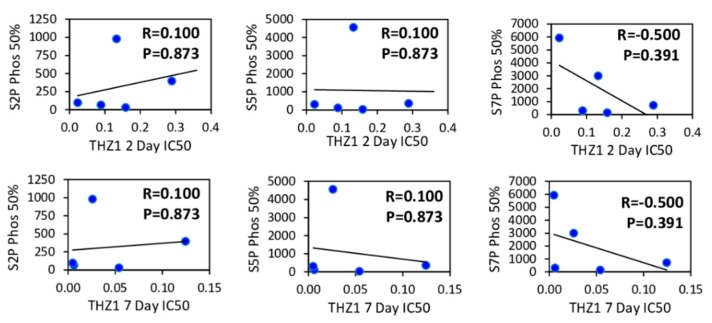
Effect of THZ1 on phosphorylation of RNA Pol II does not correlate with THZ1 sensitivity. Using densitometric analysis of RNA Pol II S2P, S5P, S7P levels the concentration of THZ1 required to decrease the phosphorylation of each protein to 50% relative to control cells was calculated and expressed as an IC_50_ value. The S2P, S5P and S7P IC_50_ values were examined for potential correlations with sensitivity to THZ1 at 2 or 7 days using bi-variant scattergraphs and Spearman rank correlations.

CDK7 plays a key role in regulating the phosphorylation of RNA Pol II and THZ1 has previously been shown to inhibit the phosphorylation of RNA Pol II at S2, S5 and S7 positions of the heptapeptide repeat comprising the C-terminal domain (CTD) in HER2+ and TNBC cells [2,17]. We tested the effects of THZ1 on the phosphorylation of RNA Pol II at S2, S5 and S7 across multiple breast cancer cell lines and found that regardless of subtype, THZ1 inhibited the phosphorylation of all three sites in a dose dependent manner (Figure 6A). Comparisons of the THZ1 concentration required to inhibit these phosphorylations to 50% of baseline levels and THZ1 growth inhibitory IC_50_ values produced no significant correlations (Figure 7), indicating that the ability of THZ1 to inhibit CDK7 enzymatic activity is not directly related to its ability to inhibit cell growth.

Previous reports have indicated that THZ1 acts as an inducer of apoptosis rather than cell cycle arrest in the TNBC cell line MDA-MB-468 [2]. Cell cycle analysis revealed that 24 h of THZ1 treatment results in G1 arrest in both a TNBC (MDA-MB-231) and an ER+ (T47D) cell line. However, after 48 h of THZ1 treatment, the ER+ cell line was still showing G1 arrest, while the TNBC cell line showed increased sub-G1 (apoptotic cell) accumulation (Figure 6B). We then compared PARP cleavage, another marker of apoptosis, in MDA-MB-231 to one of the most sensitive cell lines (SKBR3) and one of the least sensitive cell lines (JIMT-1) and found that THZ1 induced PARP cleavage only in the HER2+ SKBR3 cells (Figure 6C). This indicates that induction of apoptosis by THZ1 is not limited to TNBC.

### 3.5. Transcriptional Regulation by THZ1 in Breast Cancer

**Figure 8:**
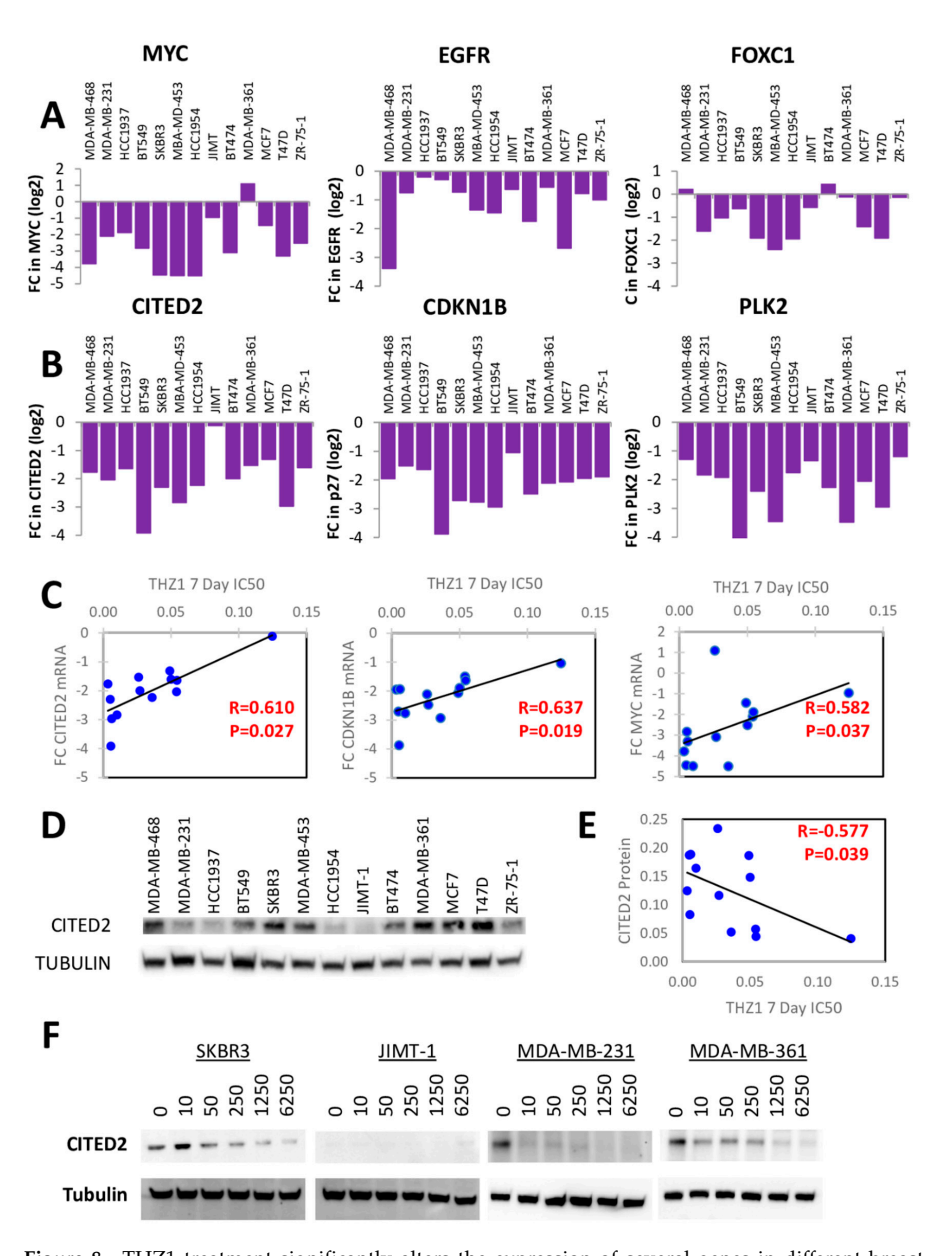
THZ1 treatment significantly alters the expression of several genes in different breast cancer cell lines. (**A**–**B**) Fold change (FC) in mRNA expression of MYC, EGFR, FOXC1, CITED2, CDKN1B, and PLK2 following treatment with 250nM THZ1 for 6 h, as compared to vehicle treated cells. (**C**) Bi-variant scatter graphs and Spearman rank correlations comparing fold change (FC) in CITED2 mRNA expression following THZ1 treatment with THZ1 sensitivity at 7 days in the cell line panel. (**D**) Immunoblotting for basal CITED2 protein expression (with α-tubulin as loading control) in cell line panel. (**E**) Bi-variant scattergraphs and Spearman rank correlations comparing CITED2 protein expression and sensitivity to THZ1 at 7 days in the cell line panel. (**F**) Immunoblotting for CITED2 protein expression (with α-tubulin as loading control) following treatment with increasing concentrations of THZ1 (nM concentrations) for 4 h.

Previously published microarray data compared the effects of THZ1 treatment on two TNBC and two ER-positive breast cancer cell lines to generate a subset of genes that were preferentially expressed in TNBC and inhibited by THZ1 [2]. This subset of genes included signaling molecules and transcription factors related to WNT, TGF-B, STAT and EGFR/MET signaling including MYC, ETS1, SOX9, TWIST1 and FOXC1. We examined the effects of THZ1 on several of these genes and found that while some of the genes including SOX9 and STAT3 were not inhibited in response to THZ1 (Figure S1), several genes including MYC, EGFR and FOXC1 were significantly inhibited by THZ1 in the majority of the cell lines tested, regardless of subtype (Figure 8A).

Therefore, we reanalyzed the microarray dataset of Wang et al. [2] to select only for genes whose expression was altered greater than 2-fold by THZ1 in all 4 cell lines. This analysis uncovered that only 5–6% of genes (1730/30724) were inhibited greater than 2-fold in these cell lines. The top 20 genes ranked by largest fold change in gene expression in response to 250 nM THZ1 in MDA-MB-468 cells are shown in Table 2. In our qPCR assays, many of these genes were either unchanged or induced, rather than inhibited, by THZ1 (Figure S2). However, we found 3 genes (CITED2, CDKN1B and PLK2) which exhibited a significant decrease in expression following THZ1 treatment in all of the tested cell lines (Figure 8B). Significant positive correlations were found between the THZ1 7-day IC_50_ values and the fold change in THZ1-induced inhibition of CITED2 (*p* = 0.027) and CDKN1B (*p* = 0.019), as well as MYC (*p* = 0.037) (Figure 8C). No significant correlations were detected for THZ1-induced changes in CITED2 or CDKN1B mRNA expression when compared to THZ1 2-day IC_50_ values, nor were any correlations found between THZ1-induced changes in PLK2 mRNA expression and either IC_50_ time point.

### 3.6. CITED2 as a Determinant of CDK7 Dependence

Transcriptional co-regulator CITED2 has recently been reported as a prognostic factor in breast cancer patients with important roles in cancer progression, chemoresistance and metastasis [22,23]. Therefore, we examined in more detail the association of CITED2 with response to CDK7 inhibition. CITED2 protein was differentially expressed across the cell line panel, but no relationship between basal CITED2 protein levels and breast cancer molecular subtype was observed (Figure 8D). However, basal CITED2 protein expression was significantly correlated with THZ1 sensitivity at 7 days (*p* = 0.039; Figure 8E). Furthermore, we found that CITED2 protein expression significantly decreased in response to THZ1 (Figure 8F). This suggests that CITED2 expression could be a predictive biomarker of response to THZ1 in breast cancer.

### 3.7. Synergy Between CDK7 and EGFR Inhibitors in Breast Cancer

**Figure 9:**
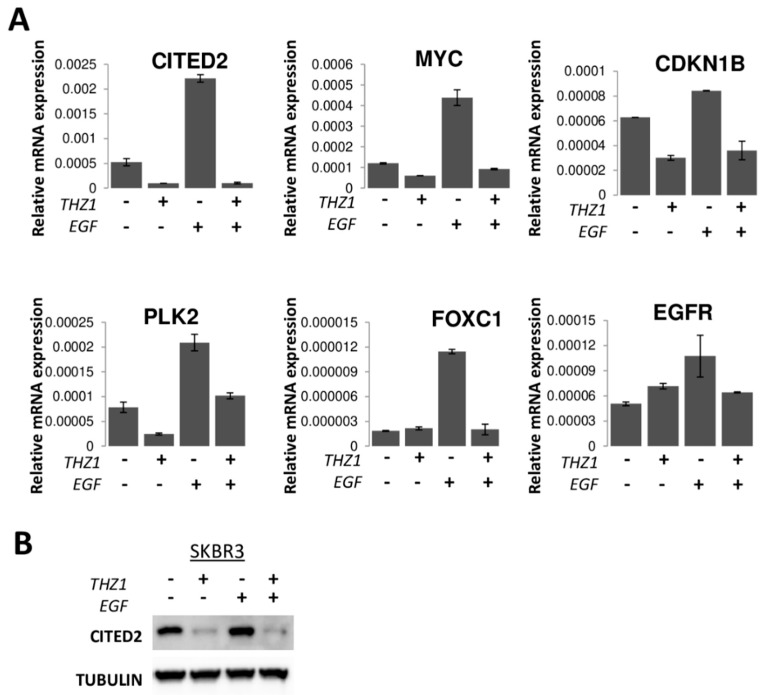
CITED2 and THZ1 sensitivity. mRNA expression of CITED2, MYC, CDKN1B, PLK2, EGFR, and FOXC1 (**A**) and CITED2 protein expression (**B**) in serum starved SKBR3 cells treated with 100 ng/mL EGF and 250 nM THZ1 for 6 h.

Most of the THZ1 sensitive genes identified here have previously been shown to be regulated by EGF stimulation or EGFR inhibition. Although CITED2 has not previously been linked to the EGF pathway, CITED2 is a downstream target of MYC [24], which is activated by EGFR signaling [25]. We hypothesized that CITED2 may be regulated in both an EGFR- and CDK7-dependent manner. We first examined whether the expression of CITED2 was regulated by EGF using the most THZ1-sensitive cell line, SKBR3. Treatment of serum-starved SKBR3 cells with EGF resulted in a 4-fold increase in CITED2 mRNA expression, and this induction was prevented by the addition of THZ1 (Figure 9A). Similarly, EGF treatment increased mRNA expression of MYC, CDKN1B, PLK2, FOXC1 and EGFR and the addition of THZ1 again blocked these EGF-induced increases (Figure 9A). EGF treatment also increased the expression of CITED2 protein in serum starved SKBR3 cells and this increase was prevented by THZ1 (Figure 9B), suggesting that CITED2 expression is EGF and CDK7 dependent.

**Figure 10:**
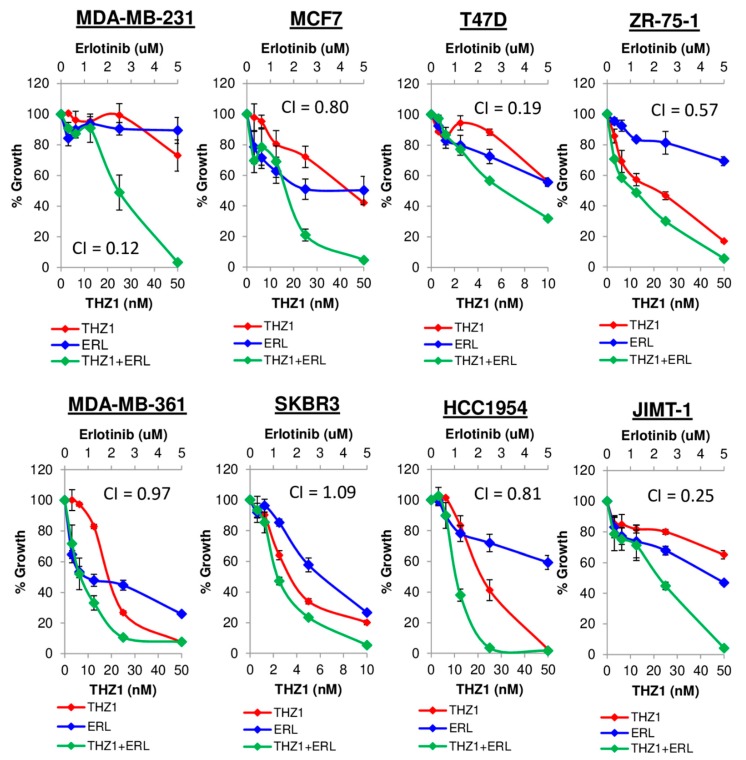
Dual inhibition of EGFR and CDK7. Cell growth curves for cell lines treated with low-dose THZ1 (0–50 nM) alone and in combination with erlotinib (0–5 µM) in fixed ratio combinations for 7 days.

Having found that many of the genes inhibited by THZ1 are regulated in an EGF-dependent manner, we hypothesized that inhibition of CDK7-mediated transcription via THZ1 could potentiate the effects of EGFR inhibition in breast cancer. To test this multiple cell lines were treated with low dose THZ1 in a fixed-ratio combination with the EGFR inhibitor erlotinib for 7 days. Synergy was analyzed using CompuSyn software to determine Combination Index (CI) values based on the drug combination principles of Chou-Talalay [20], providing a quantitative definition for additive effect (CI = 1), synergism (CI < 1), and antagonism (CI > 1) in drug combinations. THZ1 synergized with erlotinib in the majority of tested cells lines (Figure 10), with the most significant synergism occurring in TNBC MDA-MB-231 cells (CI value of 0.12). The same combination treatment resulted in additive effects in SKBR3 and MDA-MB-361 cells. These results suggest that combining CDK7 and EGFR inhibitors may be a novel therapeutic approach for the treatment of breast cancer.

## 4. Discussion

It has been suggested that TNBC [2,18] breast cancers may be uniquely dependent on CDK7. Consistent with previous reports [18], we found a strong positive correlation between high CDK7 expression and worse relapse free survival in TNBC. However, we also found strong positive correlations between CDK7 RNA expression and survival in both luminal B (rapidly growing ER+) and HER2+ breast cancer patients, the HER2+ findings consistent with a recent report [17]. In contrast to negative prognostic correlations of CDK7 RNA levels, a study that used immunohistochemistry-based CDK7 protein quantitation concluded that CDK7 protein, while elevated in breast cancers relative to normal tissues, was correlated with longer breast cancer specific overall survival [14]. Here, we found no correlation between CDK7 mRNA and protein levels in the panel of breast cancer cell lines (in contrast to excellent RNA/protein correlations for ER and HER2), explaining the discrepancy with the protein-based analysis and suggesting possible post-transcriptional regulation of CDK7 expression.

In the present study, we compared the growth inhibitory effects of THZ1 in ER+, HER2+ and TNBC cell lines, and investigated the determinants of THZ1 sensitivity across different breast cancer subtypes. We treated a panel of 13 breast cancer cell lines with THZ1 for 2 or 7 days and found that almost all of the tested cell lines were inhibited at nanomolar concentrations of THZ1, but there was still a wide range of variability of IC_50_ values within each subtype. Other studies have reported that MCF7, a widely used ER+ breast cancer cell line, was sensitive to THZ1 [26] and that HER2-expressing cancers also showed a high level of THZ1 sensitivity [17]. Hence, other factors not specifically related to breast cancer molecular subtype influence sensitivity to CDK7 inhibition.

In agreement with previous reports, THZ1 significantly inhibited the phosphorylation of RNA Pol II C-terminal domain at S2, S5 and S7 in a dose-dependent manner in all cell lines [2,9,17,18,26], confirming functional CDK7 inhibition. However, the magnitude of THZ1-induced decreases in Pol II phosphorylation did not correlate with THZ1 sensitivity. CDK7 also plays a role in cell cycle progression by acting as a CDK-activating kinase and phosphorylating multiple CDKs including CDK1, CDK2, CDK4 and CDK6 [8,27]. Previous reports examining the effects of THZ1 on TNBC showed that THZ1 induced apoptosis [2,18] and a modest accumulation of cells in G2-M phase [18]. We found that THZ1 induced G1 arrest in all the tested cell lines but only some of them showed sub-G1 accumulation or PARP cleavage, markers of apoptosis. This variability is consistent with studies reporting THZ1 effects on cell cycle arrest and apoptosis in T-ALL cells [9], G2 cell cycle block leading to DNA damage accumulation in high grade glioma [12], G2-M phase accumulation and apoptosis in MYCN-expressing neuroblastoma cells [10], and G2-M phase arrest in esophageal squamous cell carcinoma [28], as well as a study reporting no effects of THZ1 on cell cycle distribution in peripheral T-cell lymphoma [29].

To elucidate the determinants of THZ1 sensitivity, we then examined the effects of THZ1 on CDK7-regulated transcription. Wang et al. described TNBC as being exquisitely dependent on CDK7 due to an “Achilles cluster” of TNBC-specific genes regulated by super-enhancers requiring CDK7 [2]. We re-analyzed reported microarray data comparing THZ1 effects on both TNBC and ER positive breast cancer [2], with the assumption that genes which are critical to the effectiveness of THZ1 would be altered in all cell lines regardless of subtype. Although many of the genes detected as being significantly inhibited by THZ1 in the former analysis failed to show consistent inhibition across the panel of cell lines in our hands (in some cases showing increased expression compared to untreated cells), we were able to identify a small set of genes which were consistently decreased in the majority of cell lines: MYC, FOXC1, CDKN1B, PLK2, CITED2 and EGFR. Several of these genes, including EGFR, FOXC1 and MYC were reported by Wang et al to be CDK7-sensitive TNBC-enriched super-enhancer-associated genes [2]. CDK7 has previously been reported to be a therapeutic target in cancers driven by MYC [10] and THZ1 has consistently been shown to decrease the expression of MYC protein and mRNA [17,18,29,30]. Here we show that the magnitude of this decrease is positively correlated with THZ1 sensitivity, with the cell lines showing the greatest decrease in MYC mRNA expression in response to THZ1 treatment being the most sensitive to THZ1-induced growth inhibition. We also found that THZ1 decreases CDKN1B mRNA expression and that the magnitude of these decreases correlated with THZ1 sensitivity across the cell panel. MYC has been shown to facilitate the activation of CDK4/6 by inhibiting CDKN1B at the protein and mRNA level and decreases in MYC expression are usually associated with increased CDKN1B expression [31]. CDK7 inhibition also decreased EGFR, FOXC1, and PLK2 mRNA expression in most of the cell lines tested, although we found no significant correlations between THZ1 sensitivity and expression changes for these genes. CDK7 inhibition has previously been reported to downregulate EGFR in high-grade glioma [12] and PLK2 in a lung cancer cell line [30].

We were particularly interested in the finding that THZ1 inhibited CITED2 expression as this gene, not previously associated with CDK7, showed one of the strongest associations with THZ1 sensitivity. CITED2 (CBP/p300–interacting trans-activator with Glu/Asp–rich carboxy-terminal domain-2) is a non-DNA binding transcriptional co-regulator that modulates the activity of multiple transcription factors including p300/CBP, Smad2/3 and estrogen receptor [32,33,34] CITED2 is overexpressed in breast cancer compared to normal mammary tissue and CITED2 was significantly associated with increased incidence of recurrence and breast cancer-specific death of the breast cancer patients [23]. High CITED2 mRNA expression was associated with poor survival in ER positive breast cancer patients [35] and may contribute to anti-estrogen resistance [36]. CITED2 promotes proliferation, migration and resistance to chemotherapy in breast cancer [23]. In addition to finding that THZ1 significantly decreased the expression of CITED2 and that the fold change in CITED2 in response to THZ1 treatment was positively correlated with the sensitivity of the cells to THZ1, we found a significant correlation between the basal protein levels of CITED2 in the panel of cells and sensitivity to THZ1. This suggests that CITED2 may potentially be a predictive biomarker of response to THZ1.

Interestingly, the expression and/or activity of FOXC1 [37], MYC [38], and PLK2 [39] have been shown to be dependent on EGFR activity or on the expression and activity of its ligand EGF and CDKN1B has been shown to regulate EGFR expression [40]. Here, we examined if CITED2 expression was also regulated in an EGFR-dependent manner. EGF stimulation resulted in a significant increase in CITED2 expression, an effect that was inhibited by the addition of THZ1, suggesting that both EGFR and CDK7 play a role in the regulation of CITED2. (Similar results were obtained with PLK2, CDKN1B, FOXC1, and MYC). These findings suggested that targeting EGFR in combination with CDK7 may have a synergistic effect. Indeed, we found that in the majority of tested breast cancer cell lines combination treatment with erlotinib and THZ1 resulted in additive or synergistic in vitro effects on growth inhibition. These combinatorial effects were not breast cancer subtype specific, as the top three responding cell lines were TNBC (MDA-MB-231), ER+ (MCF7), and HER2+ (JIMT-1), respectively.

Our results suggest that EGFR and CDK7 combination therapy may be a novel treatment strategy for breast cancers, regardless of subtype. Tumors resistant to current clinical regimens are found in all subtypes of breast cancer, indicating a need for new treatment paradigms. As CDK7 inhibitors progress towards clinical development, identification of patients who are more likely to respond to such inhibitors becomes important. Our results suggest that CITED2 should be explored as a marker of sensitivity to CDK7-targeting drugs. Additionally, no targeted therapies for TNBC are currently available. EGFR is known to be overexpressed in up to 50% of TNBCs, but EGFR inhibitors (alone or in combination with cytotoxic chemotherapy) have proven disappointing in the clinic [41] However, a recent study demonstrated that inhibiting CDK9 sensitizes TNBC cell lines to EGFR inhibitors [42] suggesting that a combinatorial approach, specifically with CDK inhibitors, may yield an effective treatment for TNBC. This novel treatment strategy has the potential to benefit not only breast cancer patients who do not respond to current treatments, but can also be examined as a novel strategy in other cancer subtypes which has previously exhibited sensitivity to CDK7 inhibition including lung cancer, ovarian cancer, neuroblastoma, glioma, and leukemia.

## Figures and Tables

**Table 1:** THZ1 IC_50_ data in breast cancer cell line panel after 2 days and 7 days of treatment with increasing concentrations of THZ1. IC_50_ values were determined using CompuSyn software.

Cell Line	2 Day IC_50_ (µM)	7 Day IC_50_ (µM)
MDA-MB-468	0.1451	0.0034
MDA-MB-231	0.1590	0.0540
HCC1937	0.0847	0.0543
BT549	0.0937	0.0054
SKBR3	0.0230	0.0050
MDA-MB-453	0.1330	0.0100
HCC 1954	0.1580	0.0360
JIMT-1	0.2890	0.1246
BT474	0.3598	0.0270
MDA-MB-361	0.1330	0.0260
MCF7	0.0320	0.0490
T47D	0.0890	0.0061
ZR-75-1	0.1288	0.0500

**Table 2:** Genes that are altered greater than 2-fold in MDA-MB-468, BT549, T47D and ZR751 cells were ranked by the fold change in MDA-MB-468 and the top 20 genes are listed.

Gene Name	468 FC	549 FC	T47D FC	ZR751 FC
CYR61	–4.82	–2.96	–1.96	–1.81
DKK1	–4.7	–5.01	–1.09	–4.15
MYC	–4.64	–3.06	–2.68	–2.98
CITED2	–4.53	–4.12	–2.71	–2.64
FBXO5	–4.19	–4.09	–2.65	–3.01
CDKN1B	–4.11	–3.06	–2.3	–2.57
PIM3	–4.05	–2.73	–2.54	–3.96
MARS2	–4.04	–3.73	–2.3	–3.75
NEDD9	–4.03	–1.53	–3.32	–2.68
E2F8	–4.01	–3.09	–4.01	–4.01
PLK2	–3.99	–3.7	–2.48	–3.04
FADD	–3.93	–3.1	–3.29	–4.12
ELF3	–3.88	–1.11	–1.22	–1.65
MOCS3	–3.84	–4.2	–3.86	–4.1
BAMBI	–3.8	–2.97	–1.74	–3.77
ZNF217	–3.77	–3.66	–2.77	–4.13
PHLDA1	–3.71	–2.72	–3.81	–2.55
TRIB1	–3.69	–4.07	–2.31	–3.54
WEE1	–3.69	–2.95	–1.96	–3.25
ZNF627	–3.68	–3.39	–2.75	–3.38

## Supplementary Materials

The following are available online at https://www.mdpi.com/2073-4409/9/3/638/s1, Figure S1: The effect of THZ1 treatment on the expression of several key genes, Figure S2: The effect of THZ1 treatment on the expression of several key genes determined from microarray analysis, Table S1: Primer sequences used for qPCR analysis of gene expression.
