# Supplementary material for: CDK7 Inhibition Is Effective in all the Subtypes of Breast Cancer: Determinants of Response and Synergy with EGFR Inhibition
Source: Cells. 2020 Mar 6;9(3):638. doi: 10.3390/cells9030638 (PMC7140476; doi:10.3390/cells9030638)
Supplement: Supplementary file 1 [file cells-09-00638-s001.pdf]

Supplementary Materials

Table A1: Primer sequences used for qPCR analysis of gene expression

Primer	Forward sequence	Reverse sequence
CDK7	TGAGAACATGGTAATGGGGAGG	ACAGTGCTCTGCCCTAAGTT
ESR1	CAGGATCTCTAGCCAGGCAC	ATGATCAACTGGGCGAAGAG
ERBB2	GGTTCCTTCCCCTAATGGGTC	CACCCCCAAAGGCAAAAACG
MYC	CCAACAGGAACTATGACCTCGACTAC	CTCGAATTTCTTCCAGATATCCT
PLK2	TTGCGGCGTAGACTTTGTTA	AGATCTCGCGGATTATCGTC
CITED2	CATATGGTCTGCCATTTCCA	AAGGTCCCCTCTATGTGCTG
EGFR	CTGACCAAAATCATCTGTGCCC	CGTGGCTTCGTCTCGGAATT
CDKN1B	ACAGAAGAAAATGTTTCAGACGGT	CTTCTGAGGCCAGGCTTCTT
SOX9	GGAGAAACCGAGGTTGGAGG	GGAGATAGCTTGTCCGGTGG
STAT3	CACCACAAGTCCCAGTAGGG	GGAACCGACATTTGTTGGGC
TRIB1	TCAAGCAGATTGTCTCCGCC	CAAAAGGCCACAGGAGAAGC
RUNX1	GGTGGGGATGGTTGGATCTG	AACCCTGGTACATAGGCCAC
IRS-1	AAGGGGCAGCGTCACATAAA	ACTACAACCCGCTCATGTCG
BAMB1	TTACAGAGGGCTGCACGATG	GTCGTGGCTGTCACAAGTCT
ELF3	CCACCTGTGGGAGTTCATCC	CATGTCCGGCTGTATCGTGA
FADD	GCGTGGGTTTTCTCCGTACA	CTCAGACAGCCGTGAAAAGC
PIM3	ACCGCGACATTAAGGACGAA	GCGCTCACCGTCGAAGT
NEDD9	CTGTCCTCACGGGGGTTATC	TCAAGGACTCAGGCTTGGAA
DKK1	TTCCTACTGTCTTCTCCTTCGT	ATCCGGCAAGACAGACCTTC
WEE1	CGGTGAAAGCTTGGGGACTT	TTGGGGACTATCACCACTTGC
IL-8	AAATTTGGGGTGGAAAGGTT	TCCTGATTTCTGCAGCTCTGT

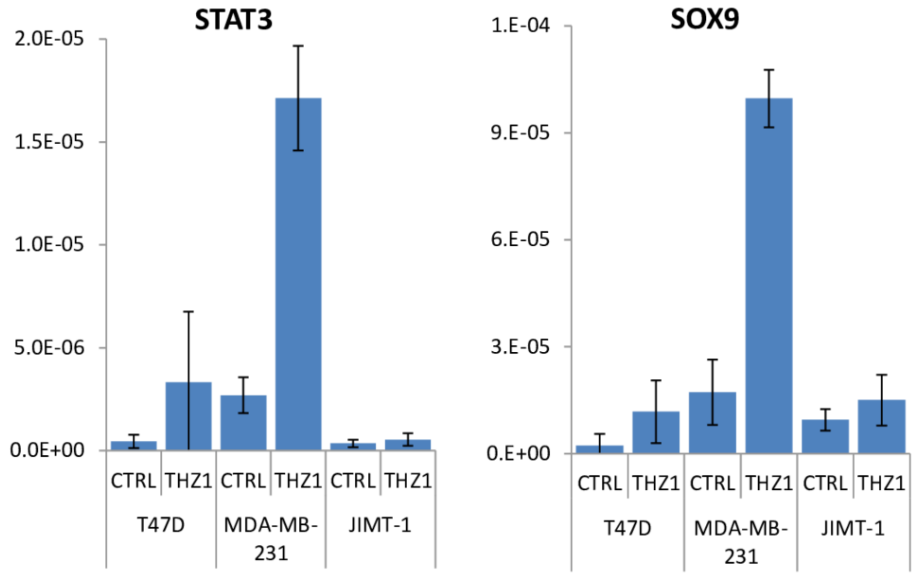

Figure A1: The effect of THZ1 treatment on the expression of several key genes. MDA-MB-231, T47D, MDA-MB-468 and JIMT-1 cells were treated with 250 nM THZ1 for 6 hours prior to RNA extraction and QPCR analysis for the expression of STAT3 and SOX9 compared to vehicle treated cells.

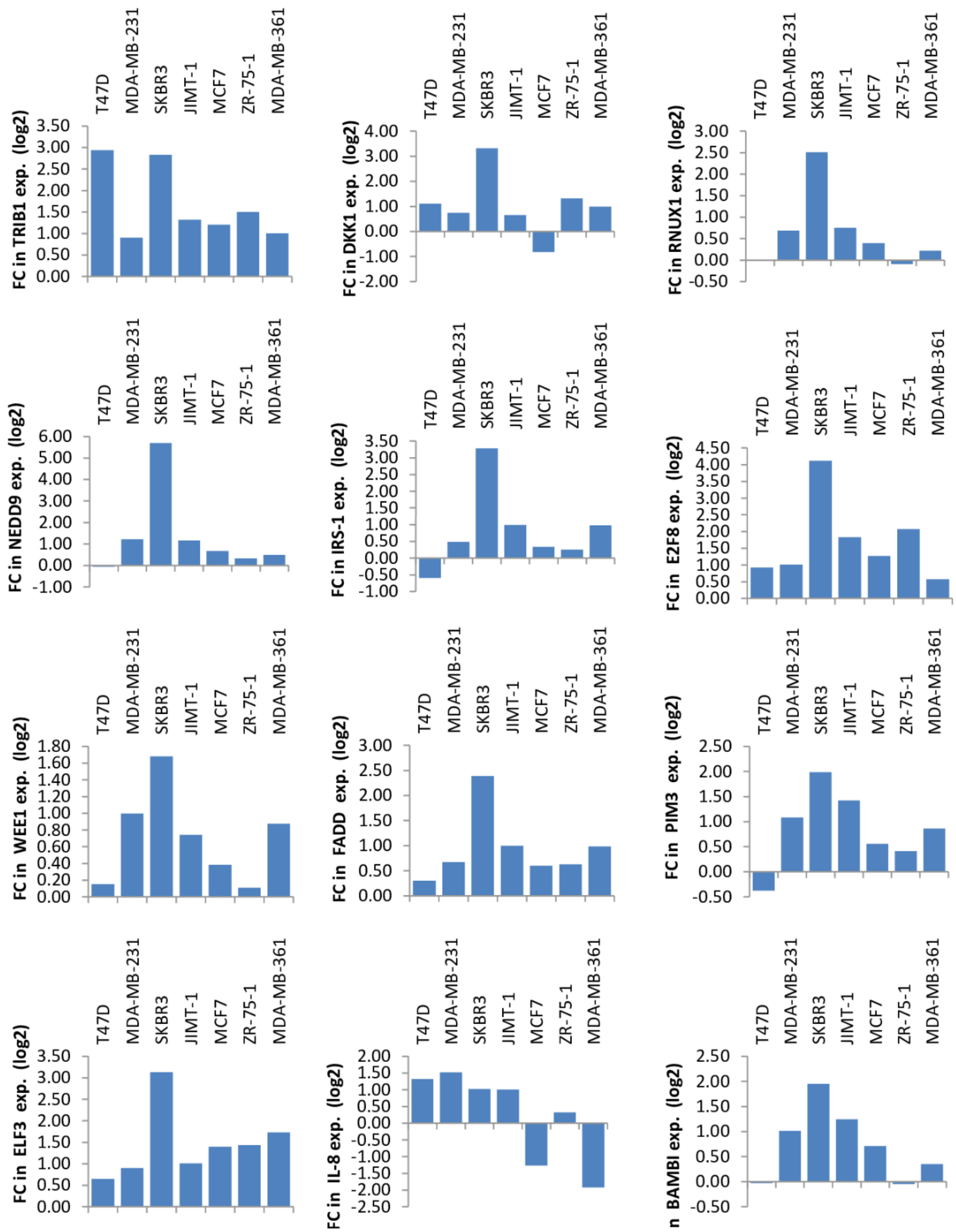

Supplemental Figure 2: The effect of THZ1 treatment on the expression of several key genes determined from microarray analysis. A panel of cell lines were treated with 250 nM THZ1 for 6 hours prior to RNA extraction and qPCR analysis for the expression of multiple genes compared to vehicle treated cells.
